# Is the Persian Version of the “Olfactory Disorder Questionnaire” Reliable and Valid?

**Published:** 2017-07

**Authors:** Maryam Jalessi, Seyed Kamran Kamrava, Elahe Amini, Farhad Rafiei, Mohammad Amin Nasouti, Naeimeh Moosavi, Mohammad Farhadi

**Affiliations:** 1 *Skull Base Research Center, Hazrat Rasoul Akram Hospital, Iran University of Medical Sciences (IUMS ), Tehran, Iran.*; 2 *ENT and Head & Neck Research Center and Department, Hazrat Rasoul Akram Hospital, Iran University of Medical Sciences (IUMS ), Tehran, Iran.*

**Keywords:** Iran, Olfaction Disorders, Quality of Life, Questionnaire, Smell

## Abstract

**Introduction:**

The Questionnaire for Olfactory Dysfunction (QOD) is a self-reporting olfactory-related quality of life questionnaire. The aim of this study was to determine the reliability and validity of the Persian version of this questionnaire.

**Materials and Methods:**

One hundred and thirteen patients with olfactory problems were enrolled in this study. The English version of the QOD was first translated into Persian. The reliability was then tested by determining the Cronbach alpha coefficient to assess internal consistency. The QOD was reviewed by a panel of experts, followed by calculating the content validity index to determine the content validity.

**Results:**

Based on the reliability analysis, the total Cronbach alpha was 0.88. The items in the “life quality” and “parosmia” domains had a good internal consistency in total, as well as in both genders and in different age subgroups. For the “sincerity” domain, however, low internal consistency was revealed (Cronbach alpha = 0.25). When questions related to the sincerity domain were omitted, the Cronbach alpha reached 0.89. The overall scale validity index for clarity and relevance were 0.88 and 0.87, respectively.

**Conclusion:**

The Persian version of the QOD seems to be a reliable and valid tool for the assessment of quality of life in patients with olfactory dysfunction. The “sincerity” domain cannot be used separately or should be substantially modified in order to be applicable to the Iranian population. However, there is no need to change the whole format of the questionnaire.

## Introduction

Olfactory impairment is a common problem. In the National Health and Nutrition Examination Survey (NHANES, 2013–2014), the prevalence of smell impairment in the US adult population (older than 40 years) was 13.5% (1). 

This impairment is considerably more prevalent in older individuals, and about one-fourth of subjects aged 50 years and older suffer from impaired olfactory function (2-4). Due to the vital role of the olfactory system, particularly against dangerous conditions, olfactory disorders can potentially affect different aspects of life.It has been clearly shown that patients who suffer from olfactory impairment tend to have a reduced quality of life because of significantly impaired personal and social daily activities and a feeling of vulnerability(5-7).Interpersonal communication may also be severely impaired (6). This is also true for overall satisfaction with life (7).

Several questionnaires have been introduced to assess general quality of life. Although most of these tools are sensitive to changes in quality of life, adopting a disease- specific questionnaire to assess quality of life (QOL) in specific patients is also needed (8,9). Olfactory loss is included in some QOL questionnaires, although the “Questionnaire for Olfactory Dysfunction” (QOD) is a specific QOL questionnaire for olfactory impairment (10,11).

The original version of the QOD included 52 questions structured as four-scale statements on life quality (LQ), sincerity (S), parosmia (P), and visual analog scales (VAS). The original form was introduced in 2005, but the number of questions was reduced to 29 in the modified version. 

A VAS consisting of five questions also accompanies the QOD. The items of the QOD are mainly focused on the domains of life that are related to the sense of taste and smell, such as social and inter-partner relationships and eating. Details of the methods for summing and calculating scores have previously been described (12).

Several studies have stated that the QOD is suitable for the specific assessment of olfactory dysfunction. However, in this study we attempted to develop a Persian version of this questionnaire and assess its reliability and validity.

## Materials and Methods

*Study population*

Patients who were referred to the olfactory laboratory of the ear, nose and throat (ENT) clinic at Rasoul Akram Hospital in Tehran, Iran during 2016 were included. These patients were referred for smell testing by the Sniff Magnitude Test (SMT). Patients were informed of the principles of the study and were then asked to complete and sign the informed consent form. All subjects were initially evaluated by an ENT specialist. Patients with mental disabilities were excluded. Demographic data including gender, age, anthropometric parameters and causes of referral were gathered. Patients were then asked to complete the QOD questionnaire before the SMT was applied for evaluation of olfactory status.

*Translation of QOD questionnaire:*

The main authors) TH and JF) were contacted by email and asked to deliver a modified English version of the questionnaire. In the first step, the English version was translated into Persian, adopting a standard methodology (10). The forward translation was performed by two independent native translators who speak the Persian language. 

The forward translations were further evaluated by 10 otolaryngologists to confirm the semantic, native, conceptual and cross cultural equivalence to the English version. The forward translation was then back translated into English by two different independent back translators who were unaware of the purposes and details of the study. A special panel including the researchers of the study reviewed the forward and the back-translated versions to produce the final Persian version of the QOD questionnaire.

*Reliability assessment*

To assess the reliability of the questionnaire, it was completed by all 113 patients referred to the clinic, and then the reliability was tested by determining the Cronbach alpha coefficient to assess the internal consistency of the QOD items. The assessment was also performed in sex and age subgroups.

*Content validity*

The QOD questionnaire was reviewed for content validity and reliability by an expert panel of 10 otolaryngologists, epidemiologists and biostatisticians for qualitative assessments. A survey was carried out into the clarity and simplicity of the items; questions were scaled based on the amount of relevancy (1=none; 2=somewhat; 3=quite; 4= highly relevant). This survey was used for the calculation of content validity index (CVI), for which the following equation was used: the sum of quite relevant and highly relevant items divided by the number of total items. Items with CVI ≥ 0.8 were considered to have good content validity.

*Statistical analysis*

To determine the internal consistency based on the Cronbach alpha coefficient, SPSS (version 16, IBM- Chicago, IL) was used for statistical analysis. To test the content validity, the CVI was calculated using its related special formula.

## Results

*Baseline* *characteristics* *of* *the study participants*

In total, 113 patients (mean age: 37.77 ± 13.81 years; range: 15–73 years; 49.6% male) were included in the study. The average of body weight was 70.94 ± 12.97 kg (range: 42–110 kg) and mean height was 169.52 ± 8.72 cm (range: 150–188 cm). The reasons for referral to the ENT clinic included accident-related trauma (57 patients ([50.44%]), dispute-related trauma (five patients [4.42%]), rhinoplasty (17 patients [15.04%]), septoplasty (17 patients [15.04%]), tumors (seven patients [6.19%]), idiopathic causes (six patients [5.30%]) and falling (four patients [3.53%]).

*Reliability of the various domains of the QOD*

Based on the reliability analysis (Table.1), the assessment based on each domain showed that the items in the LQ domain had a good internal consistency (Cronbach alpha of 0.88). The reliability remained acceptable in both men (Cronbach alpha of 0.87) and women (Cronbach alpha of 0.92), as well as in patients aged 40 years or younger (Cronbach alpha of 0.83) and older than 40 years (Cronbach alpha of 0.92). In the P domain, a good and relevant internal consistency was found overall (Cronbach alpha of 0.70), in both men and women (Cronbach alpha of 0.74 and 0.72 for ≤40 years and >40 years, respectively), and in both age subgroups (Cronbach alpha of 0.71 and 0.70, respectively). However, the Cronbach alpha was 0.25 for the S domain, revealing low internal consistency (0.24 in men and 0.27 in women) in both age subgroups (Cronbach alpha of 0.21 and 0.24 for ≤40 years and >40 years, respectively). The reliability analysis of the VAS part showed relevant internal consistency in total (Cronbach alpha of 0.88), and in men and women (Cronbach alpha of 0.87 and 0.92, respectively). However, VAS was significantly reliable only for the patients aged over 40 years (Cronbach alpha of 0.86), and not for the younger ages (Cronbach alpha of 0.60).

**Table 1:** Reliability of various domains of the Persian Version of the Questionnaire for Olfactory Disorders

**Item **	**Cronbach alpha**
All domains with VAS	0.88
ALL domains without VAS	0.86
All domains without S	0.89
All domains without S and VAS	0.86
LQ domain	0.88
S domain	0.25
VAS domain	0.76
P domain	0.70

When the VAS domain was not considered in the analysis, the reliability coefficient showed no significant change overall (Cronbach alpha of 0.86), as well as in the gender (Cronbach alpha of 0.84 for men and 0.90 for women) and age subgroups (Cronbach alpha of 0.79 for age range ≤40 years and 0.92 for higher ages). When only the LQ and P domains were considered, no significant increase in reliability of the whole questionnaire was achieved (Cronbach alpha coefficient was 0.86 in total, 0.85 in men, 0.92 in women, 0.80 in patients ≤40 years of age and 0.92 in patients >40 years). When the S domain was omitted, the reliability based on the Cronbach alpha coefficient was not significantly changed (0.89 vs 0.88).

*Content validity of the various domains of the QOD*

The validity index for clarity was 0.78 or higher for each question, and the overall scale of the validity index for clarity was 0.88. The corresponding index for relevancy was 0.78 or higher, making the overall scale of 0.87 for the relevancy. The inter-rater agreement for clarity and relevancy were 0.79 and 0.78, respectively. Based on the declaration of all 10 experts, the overall comprehensiveness of the questionnaire was scored as 95%.

## Discussion

Several studies have demonstrated some ethnical and geographical-based variations in the validity and reliability of the various questionnaires that are routinely used for the clinical assessment of patients. Various general health-related QOL questionnaires, such as the 36-Item Short From Health Survey (SF-36) or World Health Organization (WHO)-QOL questionnaire, have been widely used to assess QOL in different disease subgroups. However, disease-specific QOL assessments are also of particular interest. Neuland et al demonstrated that the QOD questionnaire was a more efficient instrument in the evaluation of olfaction-related QOL than the SF-36 (11), especially among women, showing a significant discrepancy between the two diagnostic tools. Even when disease-specific questionnaires are used, regional variations are encountered in the level of LQ (13).

The QOD is a newly developed questionnaire to evaluate the QOL in those affected by any olfactory dysfunction. However, a review of the literature shows that only a few studies have focused on the diagnostic performance of this tool and its reliability and validity to assess QOL in such patients (8,12-15). This study showed different reliability for the four components of the QOD according to gender and age. In this regard, the LQ domain had a good internal consistency in both genders and also in the different age subgroups, and thus the questions in this component do not need any modification. In contrast, the S component had insufficient reliability to assess different aspects of sincerity overall as well as in the gender and age subgroups. It should be stressed that only two patients were shown to have normal olfactory status by SMT and the others had genuine olfactory problems. As SMT is a reliable objective test for evaluation of olfaction, the questions within the S component should be omitted or greatly modified to be a reliable assessment of sincerity in our population (16,17).

In a study evaluating the reliability and validity of the Chinese version of the QOD questionnaire, Yang et al showed the Cronbach’s alpha coefficient of internal consistency of the P component was 0.473 (12), indicating low reliability in the P domain. The authors also recommended some modifications on the P section for better evaluation. As indicated in our survey, the P domain has acceptable reliability to assess QOL in both genders and different subgroups, but the same problem was encountered in the S domain. Although the S domain had a low reliability, the presence or absence of this domain did not significantly change the total reliability of the questionnaire. Thus, it seems that the QOD with the present structure can be used in our patient population. However, there are limitations on any conclusion based solely on the S domain.

## Conclusion

This Persian version of the QOD is a reliable and valid tool for the assessment of QOL in patients with olfactory dysfunction. However, when subgroups of this questionnaire are considered separately, the sincerity domain does not show reliability and should not be considered on its own for any interpretation.
